# Extended-period AOSLO imaging in the living human retina without pupil dilation: a feasibility study

**DOI:** 10.1364/BOE.531808

**Published:** 2024-08-02

**Authors:** Jiahe Cui, Maria Villamil, Allie C. Schneider, Penelope F. Lawton, Laura K. Young, Martin J. Booth, Hannah E. Smithson

**Affiliations:** 1 Department of Engineering Science, University of Oxford, Parks Road, Oxford OX1 3PJ, United Kingdom; 2 Department of Experimental Psychology, University of Oxford, Anna Watts Building, Radcliffe Observatory Quarter, Woodstock Road, Oxford OX2 6GG, United Kingdom; 3 Faculty of Medical Sciences, Newcastle University, Framlington Place, Newcastle upon Tyne NE2 4HH, United Kingdom

## Abstract

*In vivo* imaging using an adaptive optics scanning laser ophthalmoscope (AOSLO) is challenging, especially over extended periods. Pharmacological agents, administered as eye drops, are commonly used to dilate the pupil and paralyse accommodation, to improve image quality. However, they are contraindicated in some scenarios. Here, we evaluate the feasibility and reproducibility of performing AOSLO imaging without pharmacological pupil dilation over 1.5 hours with visual stimulation. Through statistical analysis and theoretical modelling using a dataset of retinal and pupil images collected from six healthy, young, near-emmetropic participants between the ages of 20–30 years, we validate that the retinal image quality does not change significantly with time in the experimental session (*p* = 0.33), and that pupil size has a strong effect on image quality but is not the only contributing factor.

## 1. Introduction

The adaptive optics scanning laser ophthalmoscope (AOSLO) is commonly used for cellular-resolution imaging of the living human retina [1–3]. As the eye constitutes the last optical element of the system, retinal image quality can be affected by a series of human factors such as refractive errors and higher order aberrations [4–6], eye movement [7,8], accommodation [9,10], pupillary response [11,12], and tear film effects [13]. During AOSLO imaging, many of these are compensated for by improving the system design and experimental protocols, such as correcting for the eye’s aberrations with closed-loop adaptive optics (AO) [14–16], keeping the participant’s head stable using a bite bar or chin rest, reducing the effects of eye movements and accommodation by presenting a suitable fixation target [17,18], and alleviating tear film effects by encouraging blinking between trials [13]. However, to remove residual accommodation effects and regulate pupillary response, dilating drops are commonly used to paralyse accommodation and enlarge the pupil during imaging [19]. The system numerical aperture (NA) of the ophthalmoscope is determined by the pupil size. Sustaining a larger pupil increases the system NA and generally leads to improved image resolution when aberrations are fully corrected [20].

However, as a topical drug dilating drops can have limitations. In many countries, the relevant pharmacological agents are only available on prescription and with appropriate medical supervision. It is also not straightforward to gain approval for drop administration or to develop reliable emergency procedures, especially when the lab is far away from the nearest eye hospital. Moreover, the participant will not be able to work or drive in the following 3-4 hours, which creates hurdles for large-scale participant recruitment. In addition to practical complications, pharmacological constraints cannot be neglected. For example, even healthy participants can have a drug intolerance [21]. Furthermore, there remains the possibility for mydriatic agents to interact with patient medication, increasing the risk factor of disease-related studies [22]. Another important consideration is that although negligible change has been observed in vessel width after administration of topical tropicamide [23] and in vessel density after administration of topical 2.5% phenylephrine and 0.5% tropicamide [24], it is not yet clear how dilating drops affect functional neurovascular physiology, such as the functioning of vascular smooth muscle, and whether it has an impact on light-evoked neurovascular response [25]. Many dynamic retinal vessel analysis studies rely on devices that necessitate widening of the pupil [26], preventing fair comparison between results with and without dilation. Last but not least, recent results suggest pupillary response to be a potential biomarker for cardiovascular [27] and neurodegenerative [28] diseases. It would be interesting to investigate how this relates to candidate retinal biomarkers, but such studies would require imaging with a natural pupil. Therefore, it would simplify imaging protocols, facilitate participant recruitment, and promote disease-related studies, if single cone photoreceptors could be resolved with high reproducibility during AOSLO imaging without dilating the pupil.

In practice, psychophysical experiments performed with an AOSLO typically last for several hours depending on the number of trials. This is usually split into multiple sessions spanning multiple days, but one session can still take up to two hours. Imaging commonly takes place in a dark room while completing a repetitive task, which makes it especially important to maintain stable imaging over long periods.

In this work, we performed a dedicated study to evaluate the feasibility and reproducibility of performing AOSLO imaging without pupil dilation over an extended period. We presented a full-field time-varying intensity-modulated visual stimulus to actively drive pupillary response while simultaneously recording images of the pupil and retina. We analysed how pupil diameter and retinal image quality changed with time over a 1.5-hour experiment, how the two metrics correlated, and how the pupil diameter changed with the intensity-modulated background. Finally, to appreciate the extent to which other factors beyond pupil size affected image quality, modulation transfer functions (MTFs) were modelled using measured pupil diameters and compared with experimental results. Although prior works have suggested the possibility of performing cone-resolved imaging without pupil dilation [29–31], and especially at peripheral regions [32], to the best of our knowledge, this is the first study that rigorously evaluates the feasibility and performance of AOSLO retinal imaging over extended periods with a natural pupil. We hope this work can provide guidance for future studies, especially when dilating drops are contraindicated for medical or research reasons.

## 2. Methods

### 2.1 Participants

Six healthy young participants between the ages of 20–30 years participated in the study, five of which had normal uncorrected visual acuity. One participant (0010) self-reported –0.5 diopters spherical and –0.5 diopters astigmatism; this participant was tested to have a visual acuity of 20/30 on a Snellen eye chart without correction and was imaged with no spectacles. All participants signed formal consents before the experiment. This study was approved by Central University Research Ethics Committee (Reference: R68805/RE003).

### 2.2 System setup

**Fig. 1.**
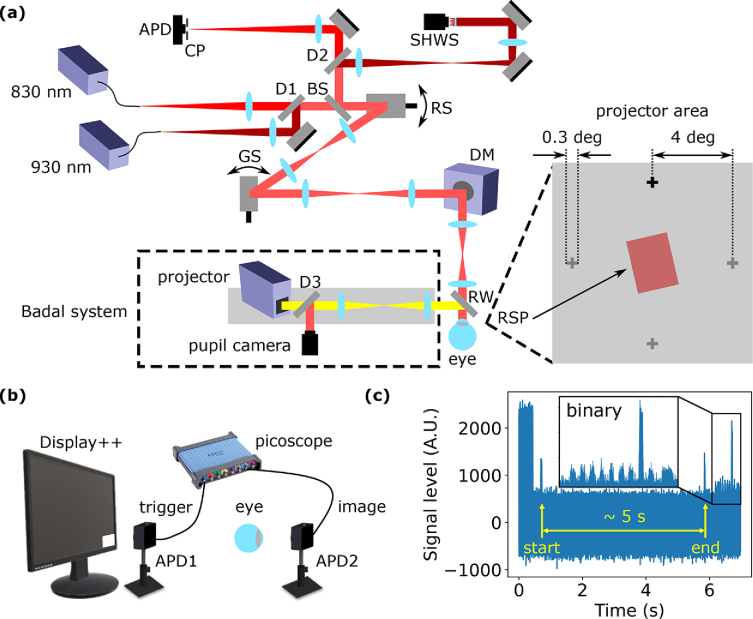
System setup and experimental design. (a) Simplified schematic diagram of custom-built AOSLO (left) and design of visual stimulation with overlaid RSP (right). A single fixation cross appeared at one of the four visual fields for any given trial, as depicted by the grey candidate crosses and black active cross. The RSP was visible and tilted 14^∘^ counterclockwise relative to the vertical axis but participants were instructed to fixate on the cross. APD: avalanche photo-diode; BS: beam-splitter; CP: confocal pinhole; D: dichroic mirror; DM: deformable mirror; GS: galvo scanner; RS: resonant scanner; RSP: raster scan patch; RW: reflective window; SHWS: Shack-Hartmann wavefront sensor. (b) Conceptual diagram of electronic synchronisation between pupil camera and AOSLO image acquisition that leverages on the Display++ and APD1 for triggering. (c) Example of a full 7-second photo-diode trace from APD1 during image acquisition.

Experiments were performed on a custom-built AOSLO. Figure 1(a) illustrates the system setup for this study. An 830 nm super-luminescent diode (SLD, Thorlabs, SLD830S-A20) was used for imaging in the near-infrared (NIR) and a 930 nm SLD (Thorlabs, SLD930S-A40W) was used for wavefront sensing. A resonant scanner (RS, Electro-optical Products Corp, SC30) scanned at 14 kHz along the fast axis and a galvanometer scanner (GS, Cambridge Technologies, 6810P), which was synchronised to the resonant scanner, provided a frame rate of 30 Hz. Closed-loop adaptive optics (AO) was performed at 10 Hz using a large stroke deformable mirror (DM, Alpao, DM97-15). A two-Airy disk diameter confocal pinhole (CP) was placed before a variable gain avalanche photo-diode (APD, Thorlabs, APD430A/M) for confocal detection and a custom-built Shack-Hartmann wavefront sensor (SHWS) comprising of a microlens array (Edmund Optics, 64-476) and an NIR-enhanced CMOS camera (Ximea, MQ022RG-CM) was used for wavefront sensing. The visual stimulus was presented via a projector (Texas Instruments, DLP LightCrafter 4500) in Maxwellian view and coupled into the eye through a Badal system. The projector area was centred on the raster scan patch (RSP) and spanned a field of view (FOV) of $11^{\circ }\times 11^{\circ }$ . An NIR-enhanced CMOS camera (pupil camera, Ximea, MQ042RG-CM) was used to monitor the participant’s pupil response at 10 Hz (limited by internal buffer) using back-scattered NIR light.

### 2.3 Experimental design

Room lights were turned off during experiments and eyes were dark adapted for at least 10 minutes before imaging. An eye patch was used to cover the fellow eye. To evaluate how the change in pupil size affects retinal image quality, a greyscale time-varying full-field sinusoidal intensity modulation was applied on the projector to actively drive pupillary response. Luminance oscillated between 0.5–25 cd/m^2^ as measured through perceptual matching with a Display++ LCD monitor (Cambridge Research Systems) that was calibrated using a SpectraScan PR-670 (JADAK). The oscillation frequency was set to 0.5 Hz to maximise pupil response [33] and a maximum luminance of 25 cd/m^2^ ensured coverage of photopic vision. For each trial, a 0.3^∘^ fixation cross appeared at 4^∘^ eccentricity relative to the centre of the RSP at one of the four visual fields to control for variation in retinal reflectivity at a single location between participants, also illustrated in Fig. 1(a). The RSP was visible during experiments, and participants were instructed to fixate on the cross at all times.

There were 5 blocks of data collection, each with 32 trials (8 for each visual field). To minimise order effects, the field order in which crosses were presented within each block was determined by dividing them into 8 identical groups that contained one of each instance, and performing a random shuffle for each group. The experiment was auto-paced following a series of beeping sounds that contrasted in length and tone to instruct the participant: 1) a long beep of tone 1 to mark the beginning of a trial; 2) three short beeps of tone 1 to remind the participant to blink their eyes and prepare; 3) a short beep of tone 2 that marks the beginning of image acquisition; 4) another short beep of tone 2 to mark the end of image acquisition; 5) a long beep of tone 3 to mark the end of a trial. There was a 5-second interval between trials and all participants were invited to take a 2-3 minute break in a lighted environment between blocks. The full experiment took approximately 1.5 hours.

### 2.4 Electronic synchronisation

Two streams of images were recorded for the experiment: pupil images from the pupil camera and retinal images from the AOSLO. AOSLO image acquisition was controlled through a separate custom-written Python software and live streamed to a compact PC-controlled oscilloscope (picoscope, Pico Technology, Picoscope 4824A) [3] for data recording.

To synchronise pupil and retinal image acquisition, we developed a passive triggering technique that relied on the Display++ and a UV-enhanced variable gain APD (APD1, Thorlabs, APD430A2/M), as illustrated in Fig. 1(b). The Display++ was controlled through Psychopy software (Open Science Tools Ltd.) [34] to present triggering signals in the form of greyscale flashes differing in intensity and duration. Within each trial, a white flash first initiated AOSLO acquisition, followed by signals that marked the start and end of pupil camera acquisition, and a binary trace that encoded the block and trial number at 16.7 Hz. Signals were collected by APD1 and live streamed into the same picoscope device as the AOSLO retinal imaging signals, which inherently provided synchronisation between different channels. To unambiguously decode the triggering signals, the peak of each flash was found after the signal was bandpass filtered to remove low frequency intensity variation and high frequency noise such that the 16.7 Hz binary signal could be precisely extracted. Peak values in the filtered signal between 0.75–1 of the highest peak value (corresponding to the white flash) were classified as binary ‘1’ and those lower than 0.25 as binary ‘0’.

The pupil camera recorded for a 5-second period (50 images) and the AOSLO for an overlapping 7 seconds (210 images) to include the binary trace and ensure full coverage of the pupil camera recording. An example of the full 7-second photo-diode trace is provided in Fig. 1(c). Visual stimulation and pupil camera acquisition were controlled from Psychopy through the same custom-written software and precisely synchronised. The intensity-oscillating visual stimulus delivered through the projector was turned on at the same initial phase for each trial immediately before pupil camera acquisition.

### 2.5 Image processing

**Fig. 2.**
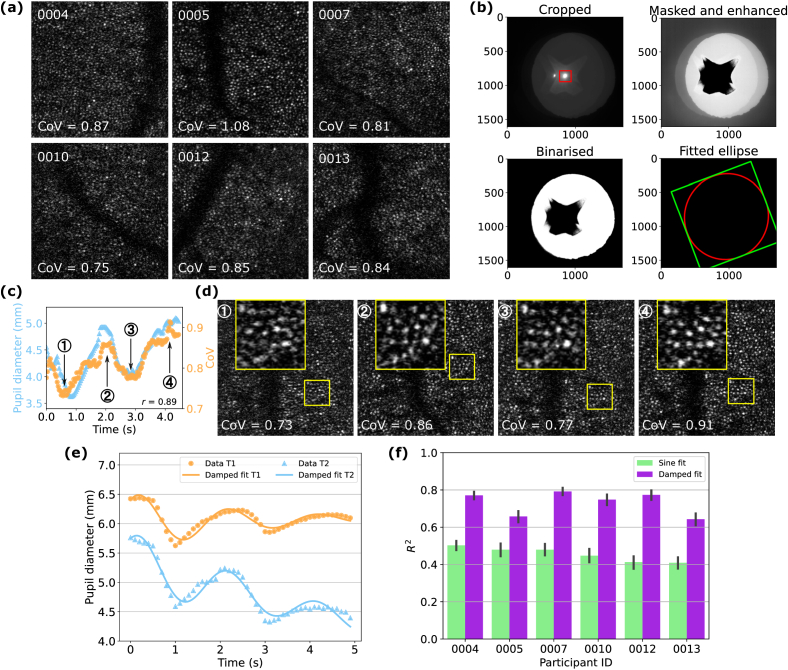
Image processing and analysis. (a) Example raw images for each participant with corresponding CoV values. Image size: $1.25^{\circ }\times 1.25^{\circ }$ . (b) Pipeline for pupil diameter extraction. First Purkinje reflection in red box used as reference for cropped image. (c) Raw image CoV trace and pupil diameter trace for one trial from participant 0005, resulting in a Pearson correlation coefficient of 0.89. (d) Raw images corresponding to the local minima and maxima of the CoV trace in (c) with magnified views of the same retinal patch at each time point considering fixational eye movements. (e) Example pupil diameter traces fitted using the proposed damped sinusoidal model for trials that show amplitude damping (T1) and a change in mean pupil diameter (T2). (f) Comparison of $R^{2}$ values using a standard sine fit (green) and the proposed damped fit (purple) for all participants. Error bar: 95% confidence interval.

Raw retinal images were desinusoided and analysed without denoising or averaging. The coefficient of variation (CoV) was used as a general image quality metric, as defined by

(1) $$\text{CoV}=\frac{\sigma }{\mu },$$

where *σ* and *μ* denote the image standard deviation and mean, respectively. Figure 2(a) demonstrates an example raw image for each participant with their corresponding CoV values.

The pupil diameter was extracted following the pipeline outlined in Fig. 2(b). To reduce computational demand, the full pupil image was cropped. Under the imaging conditions for this study, the first Purkinje reflection was approximately centred on the pupil and provided a strong reflection that could be used to localise the pupil. A cropping window which was centred on the Purkinje image and large enough to contain the full pupil area could therefore be determined. A dim secondary image of the pupil and Purkinje reflection was introduced by the reflective window (RW) immediately in front of the eye due to the absence of an anti-reflection coating. In practice, the Purkinje image with a larger contour was used as reference, highlighted in the red box. Then, the highly reflective region was masked according to an empirical threshold and the whole image was brightness and contrast enhanced. Finally, the image was binarised and an ellipse was fitted to the pupil. A minimum contour area was set to ensure correct ellipse fitting, and the pupil diameter was calculated by averaging the major and minor axes. Pupil images containing blinks were detected using Canny edge detection prior to cropping images and removed by setting the pupil diameter to NaN, together with images that failed to detect a first Purkinje reflection. After pupil diameter extraction, traces for each trial were cleaned to account for fitting errors that manifested as sudden large jumps between continuous data points and did not resemble plausible biological reaction.

### 2.6 Image correlation

To understand the relationship between raw image CoV values and pupil diameter as a result of sinusoidal intensity modulation, the Pearson correlation was calculated between the two traces. It should be noted that the triggers marking the start and end of pupil camera acquisition were displayed before and after the actual image capture. To compensate for this time difference, the maximum correlation *r* was found for each trial through an iterative search process that systematically added an increasing number of frames to the ‘starting’ AOSLO image and subtracted an increasing number of frames from the ‘ending’ AOSLO image. To account for differences in the acquisition rate between the raw image CoV trace (30 Hz) and pupil diameter trace (10 Hz), the latter was interpolated according to the actual number of AOSLO frames with a piecewise cubic polynomial using natural boundary conditions and no extrapolation. Figure 2(c) provides an example of a raw image CoV trace and pupil diameter trace for one trial from participant 0005, showing a strong correlation of r=0.89 . Raw images corresponding to the local minima and maxima of the CoV trace suggest that despite the oscillation in image quality, even at time point ① with the lowest CoV value, single photoreceptors were still clearly visible, as shown in Fig. 2(d). A magnified view of the same retinal patch at each time point is provided considering fixational eye movements.

### 2.7 Parametric fitting

A parametric model was used to extract metrics on how the pupil diameter changed and affected image quality within one trial. This was achieved by fitting a sinusoid to the pupil diameter trace using linear least squares regression. We adopted a damped sinusoidal model in the form of

(2) $$D\left(t\right)=Ae^{-\lambda t}\cos\left(\omega t+\varphi \right)+Bt+C,$$

where D(t) is the instantaneous pupil diameter at time *t*, *A* is the initial amplitude, *λ* is the amplitude decay rate, *ω* is the angular frequency, *φ* is the initial phase at t=0 , *B* is the gradient of the mean pupil diameter, and *C* is the mean pupil diameter at t=0 . During regression, the angular frequency was set as a fixed parameter corresponding to the 0.5 Hz intensity modulation. Initial values used for other free parameters are given in Table 1. This model provided a more accurate resemblance to the data compared to a standard sinusoidal model, as demonstrated in Fig. 2(e) for trials that observe noticeable amplitude damping (example T1) or change in mean pupil diameter (example T2). This was further validated in Fig. 2(f) by comparing the *R*-squared (*R^2^*) values between a standard sine fit (green) and the proposed damped fit (purple), where the latter resulted in a higher *R^2^* value for all participants. Error bars indicate a 95% confidence interval.

**Table 1.** Initial values for free fitting parameters^*a*^

Parameter	A	λ	φ	B	C
Value	$3\sigma_{t}/\sqrt{2}$	0.5	π/8	0	$\mu_{t}$

^*a*^ $\sigma_{t}$ : within-trial standard deviation; $\mu_{t}$ : within-trial mean.

### 2.8 Modelling of the modulation transfer function

Although changes in pupil size are a major limiting factor when imaging without pupil dilation, there could be other factors affecting image quality, such as accommodation, residual aberrations of the eye, tear film effects, detector noise, etc. To determine whether or not there was a further reduction in image quality contributed by these factors beyond what is expected by diffraction at the measured pupil size, we generated simple models of the eye’s modulation transfer function (MTF). Considering accommodation to be a prominent contributor, we modelled the ensemble of this reduction as different amounts of effective defocus during MTF analysis and compared the model with experimental results.

The model took into account measured pupil diameters, a typical back focal length of 17 mm, and an average vitreous humour refractive index of 1.33. Peak-to-valley (p-v) amplitudes of Zernike defocus between 0% and 100% of the imaging wavelength were considered with 1% increments. A double-pass incoherent imaging model was used where the effective detection point spread function (PSF) was calculated as the modulus squared of the single-pass intensity PSF. The MTF was then obtained by taking the modulus of the Fourier transform of the effective detection PSF. Within one retinal image, two cones at random locations were selected as central cones, and the profile plots between each of these cones and their six adjacent cones were extracted. Two metrics were calculated from the profile plots: 1) inter-cone distance *d*, and 2) Michelson contrast *C*. Within each image *n*, the mean inter-cone distance $d_{n}$ and mean Michelson contrast $C_{n}$ were calculated as:

(3) $$d_{n}=\frac{\sum_{i=1}^{L}\sum_{j=1}^{M}d_{ij}}{L\times M},$$

and

(4) $$C_{n}=\frac{\sum_{i=1}^{L}\sum_{j=1}^{M}C_{ij}}{L\times M},$$

where *i* denotes the *i*-th sampled location, *L* is the number of locations sampled in each image, *j* denotes the *j*-th measurement at a single location, and *M* is the number of measurements at a single location. Finally, $1/d_{n}$ and $C_{n}$ were taken as the critical spatial frequency and contrast, and compared with the defocused MTFs to identify the amount of effective defocus that best matched with experimental results considering all other factors that affected image quality. Cones were manually labelled in ImageJ and profile plots were plotted in Matlab.

## 3. Results

### 3.1 Image quality and pupil diameter reproducibility

**Fig. 3.**
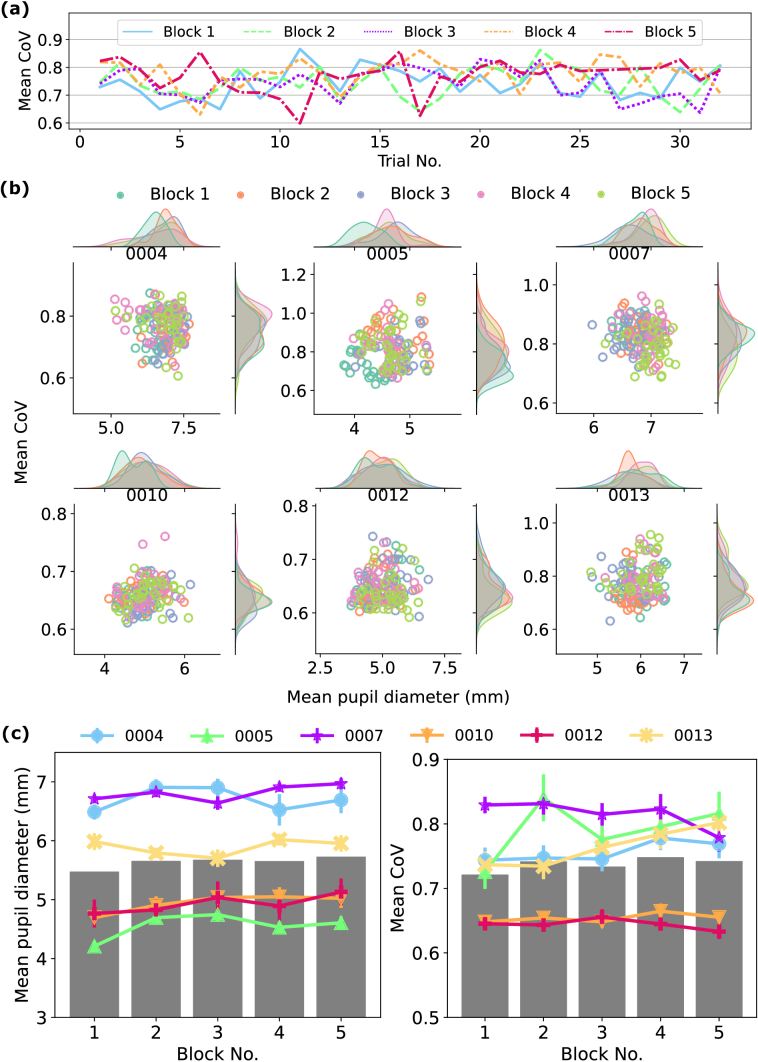
Results of image quality and pupil diameter reproducibility. (a) Example time series plot of mean CoV against trial number for participant 0004. Each coloured curve represents one block of the experiment. (b) Joint plots illustrating the effect of block number on mean pupil diameter and mean image quality. (c) Summary of one-way repeated measures ANOVA. Each coloured curve represents one participant, error bars indicate a 95% confidence interval across 32 trials of one block, and grey bars take the mean of each block over all participants. Left: Effect of block number on mean pupil diameter. Right: Effect of block number on mean image quality.

To evaluate the feasibility of performing AOSLO imaging over extended periods without pupil dilation, we were interested in understanding how the mean image quality and mean pupil diameter varied across the 1.5-hour imaging period. For each trial, the mean CoV was calculated over the frames that corresponded to a maximum correlation with the pupil diameter trace, as elaborated in Section 2.6, and the mean pupil diameter was taken over the full pupil diameter trace. Figure 3(a) shows an example time series plot of mean CoV against trial number for participant 0004. Each coloured curve represents one block of the experiment. Results showed no noticeable separation between blocks, nor a trend to incline or decline with trial number within one block, suggesting good between- and within-block imaging reproducibility. To further analyse the distribution of mean image quality and mean pupil diameter, joint plots were generated for each participant, as shown in Fig. 3(b). In general, data points were evenly distributed across blocks without noticeable local clustering. The histograms slightly varied in shape between participants and some showed a separation, such as that on mean pupil diameter for participant 0004, but there was no trend seen in the distribution across participants.

As a more rigorous analysis of how imaging performance changed over the 1.5-hour experiment, ANOVAs were performed in Matlab. Pupil diameter and CoV were treated as two dependent variables, and were tested separately using one-way repeated measures ANOVA with block number (u=5) as the within-subject factor. For each participant (n=6) , the mean pupil diameter and mean CoV were taken over each trial, then over each block. Sphericity was tested and either Greenhouse-Geisser (G-G) or Huynh-Feldt (H-F) correction was used depending on the values of $\hat{\epsilon }$ and $\tilde{\epsilon }$ as compared to 0.75 . Figure 3(c) provides a summary of the results, where each coloured curve represents one participant and error bars indicate a 95% confidence interval across 32 trials of one block. Grey bars take the mean of each block over all participants. Results showed no significant effect of block number on mean pupil diameter (p=0.14,n=6,G-G) or mean image quality (p=0.38,n=6,H-F) , confirming the high degree of imaging reproducibility across blocks, and validating the feasibility to image over extended periods without pupil dilation.

### 3.2 Image quality–pupil diameter correlation and pupillary response

**Fig. 4.**
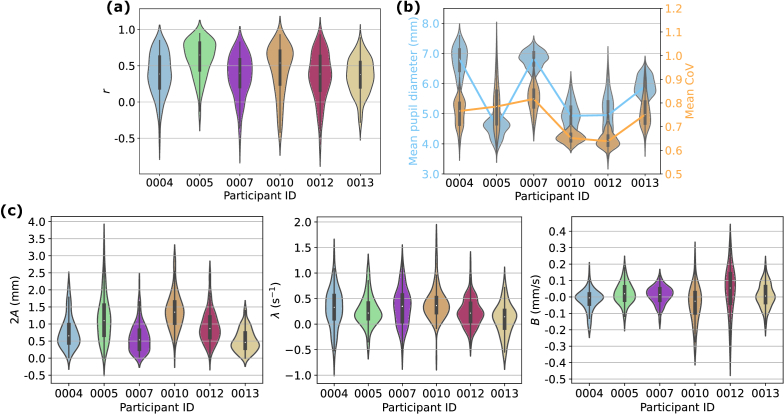
Results of image quality–pupil diameter correlation and pupillary response for each participant. (a) Distribution of within-trial correlation between pupil diameter and CoV. (b) Distribution of the mean pupil diameter (blue) and mean CoV (orange) taken over each trial. (c) Distributions of three critical metrics extracted from the parametric model describing pupillary response. Left: initial peak-to-valley amplitude response (2*A*). Middle: amplitude decay rate (*λ*). Right: gradient of mean pupil diameter (*B*).

We then looked at the effect of pupil diameter change on image quality to identify optimal imaging conditions for a natural pupil. Pearson correlation coefficients (*r*) were calculated according to Section 2.6 for all trials obtained from each participant. The plot in Fig. 2(c) exemplifies a case of high within-trial correlation. Further analysing the ensemble of *r* values for each participant led to the distributions presented in Fig. 4(a), suggesting in general a positive correlation with a median value around 0.5. However, there exist trials showing low correlation (*r* values), indicating that changes in the pupil diameter did not account for a high proportion of the variance in image quality for those particular trials. We then took the mean pupil diameter and mean CoV for each trial and evaluated the two distributions for each participant, as demonstrated in Fig. 4(b). Results showed that in general, participants with a smaller pupil size exhibited lower image quality and vice versa, which aligns with our expectations. However, this is with the exception of participant 0005, who delivered the second highest image quality despite having the smallest pupil. We note that for this participant we were able to visualise clear cone photoreceptors even without AO.

The results above validated the within-trial and within-participant correlation between image quality and pupil diameter, suggesting that optimal imaging would be performed by keeping light levels dim and steady to maintain a larger pupil. With this in mind, we finally assessed how pupil size changed with the sinusoidal intensity modulation in each trial using the parametric model described in Section 2.7. Three parameters were analysed: *A*, *λ*, and *B*, which in combination describe how well the pupil responded to the intensity-oscillating stimulus during the 5-second imaging period. Figure 4(c) summarises the distribution of the three parameters for each participant. Figure 4(c):left shows that for a 0.5–25 cd/m^2^ intensity variation, the initial p-v amplitude response (2*A*) sees a median value ranging between 0.5 mm and 1.5 mm across participants. This is followed by positive amplitude damping for all participants with a median exponential decay rate of around 0.25 s^−1^, as indicated in Fig. 4(c):middle, corresponding to a 71.4% decrease in amplitude after the 5-second period. Finally, the mean pupil diameter is shown to either incline or decline with time (Fig. 4(c):right), in extreme cases reaching a gradient of –0.5 mm/s (participant 0012), but tends to evenly distribute around zero.

### 3.3 Image quality reduction beyond the diffraction limit

**Fig. 5.**
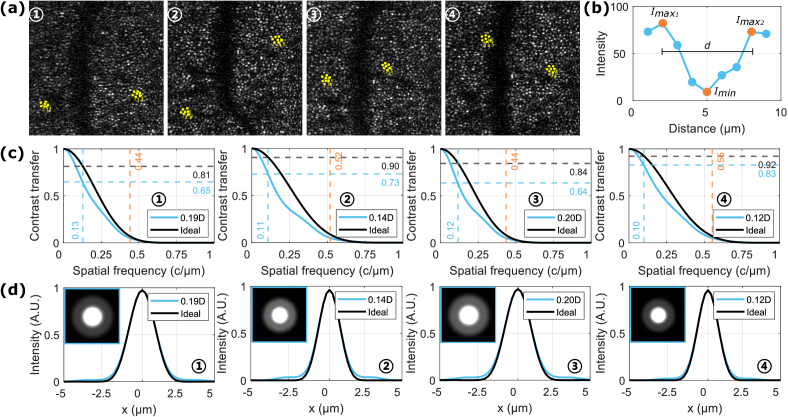
MTF modelling results of effective defocus considering reductions in image quality beyond diffraction at the measured pupil size. (a) Cones sampled for analysis in each image, as marked by yellow dots. (b) An example profile plot drawn between a central and an adjacent cone. (c) Modelled MTFs for each image in (a). Black curve: ideal MTF corresponding to the measured pupil diameter. Blue curve: MTF corresponding to the effective amount of defocus considering the ensemble of other factors beyond pupil size. Blue dashed line: critical values of $1/d_{n}$ (vertical) and $C_{n}$ (horizontal). Black dashed line: pupil size-limited contrast at $1/d_{n}$ . Orange dashed line: spatial frequency cutoff at Rayleigh resolution limit for the defocused MTF. (d) Normalised cross-sectional views of the ideal PSF (black) and defocused PSF (blue). Inset: 2D profile of defocused PSF saturated above 10% maximum intensity for visualisation purposes.

The above analysis focused on the effect of pupil size on image quality. To evaluate whether there was a further reduction in image quality contributed by other factors beyond what is expected by diffraction at the measured pupil size, in particular accommodation, the effective amount of defocus was determined for the four images from one example participant in Fig. 2(c) through MTF modelling, as outlined in Section 2.8. These images corresponded to a pupil diameter $D_{n}$ of 3.98 mm, 4.85 mm, 4.04 mm, and 5.00 mm, respectively. Yellow dots in Fig. 5(a) mark the cones that were used for analysis. Figure 5(b) depicts a typical profile plot between two cones, where $d_{ij}$ was determined as the distance between local maxima $I_{max_{1}}$ and $I_{max_{2}}$ , and $C_{ij}$ was calculated as the mean Michelson contrast of the two cones in each plot.

Black curves in Fig. 5(c) illustrate the ideal MTF corresponding to the measured pupil diameter $D_{n}$ . Measured values of $1/d_{n}$ and $C_{n}$ did not fall on these curves, suggesting that pupil size was not the only limiting factor. Blue curves indicate the MTF that corresponds to the effective amount of defocus present considering $d_{n}$ , $C_{n}$ , and $D_{n}$ . Defocus is expressed in units of diopters (D). Blue dashed lines represent the critical values of $1/d_{n}$ (vertical) and $C_{n}$ (horizontal), black dashed lines mark the contrast that would have been achieved at $1/d_{n}$ if pupil size were the only factor affecting image quality, and orange dashed lines mark the spatial frequency cutoff for the defocused MTF at Rayleigh resolution limit. Results showed that the images contained between 0.12D–0.20D of effective defocus, leading to a narrower MTF and lower contrast support. This is regardless of pupil size and could have primarily originated from residual accommodation after closed-loop correction, especially in the case of a small pupil contributing to fewer effective Shack-Hartmann spots. The spatial frequency cutoff was on the scale of foveal cones (∼2.5μm) , but being at the contrast limit means that it would be difficult to reliably image foveal cones in a naturalistic setting. To further appreciate how this is reflected in the effective detection PSF, Fig. 5(d) provides normalised cross-sectional views of the defocused (blue) and ideal (black) PSF, as well as 2D profiles of the defocused PSF saturated above 10% maximum intensity for visualisation purposes. Slight broadening of the $1/e^{2}$ width is seen at higher defocus amplitudes in the cross-sectional views, also reflected through the increasingly visible side-lobes within 2D profiles. The full width at half maximum has not been noticeably affected. This validates that at retinal locations where the inter-cone distance is relatively large, such as in this work, image quality will not be substantially compromised. However, with a smaller inter-cone spacing, the increased power in side-lobes will have a stronger effect on resolution and contrast.

## 4. Discussion

Results in this work validated the reproducibility of AOSLO retinal image quality over a 1.5-hour period without pupil dilation. A strong positive correlation was shown between changes in the image quality and pupil size within trials. This was also demonstrated between the mean image quality and mean pupil size achieved from each participant. The initial pupillary response amplitude varied between participants, and a general trend was seen for the response to adapt to the intensity modulation over the 5-second period as indicated by positive damping. Through MTF modelling we were convinced that there were other factors affecting retinal image quality in addition to pupil size, most likely residual accommodation, which resulted in an effective defocus of between 0.12D–0.20D for the examined images. This is approximately double the defocus amplitude expected at diffraction-limited performance for the same pupil diameters according to the Maréchal criterion [35].

We would like to provide some thoughts on the interpretation of this study. First, a full-field intensity modulation was applied to purposefully drive pupillary response and analyse its effect on retinal image quality. Results suggested that the best image quality would be delivered by keeping light levels dim and steady to maintain a larger pupil. The same full-field intensity modulation is generally not recommended for imaging with a natural pupil unless there is a particular reason to do so. Second, this work represents a case study of imaging at $4^{\circ }$ eccentricity. Results showed great potential for stable and good quality imaging to be performed at this retinal location without a dilated pupil. However, MTF analysis suggests that the same imaging performance would be difficult to achieve at lower eccentricities closer to the fovea. Third, we note that the good degree of reproducibility may have benefited from elements of the experimental protocol. Importantly, we kept each block of the experiment to below 15 minutes and strictly adhered to asking the participant to take a 2-3 minute break in a lighted environment between blocks. In addition, the instructive beeping sounds throughout each trial not only helped participants precisely identify the time points that required their active input, both in terms of eye preparation and image acquisition, but also notified them of when to anticipate a stimulus onset. The first element can be easily adapted to many different experiments. The second element, however, will only be valid when: 1) the time point of stimulus onset can be made available to the participant; 2) there are not too many stimuli to be presented in a trial to cause confusion; 3) the time interval between each stimulus is sufficiently long. It should also be mentioned that we did not observe strong defocus effects in this study. We sought to minimise changes in accommodation through task design, which included a fixation target, and closed-loop AO correction. The latter seemed to deal well with remaining effects of accommodation but, as the SHWS only provides information of the residual aberration after DM compensation, the actual accommodation magnitude is unknown.

Finally, we point out some limitations of this work in view of future studies. First, the results presented here are from a small number of healthy young participants. Future studies should encompass a broader age range and potential patient cohorts, considering that non-invasive imaging is valuable for disease-related studies. Second, manual cone labelling limited the number of participants, images and locations that could be analysed for MTF analysis. The results presented in Section 3.3 demonstrate the viability of using the method to understand the contribution of other factors affecting image quality beyond pupil size. Future work will develop a more automated and integrated pipeline to expand its applicability and throughput. Third, the current experiment did not set up an active participant response task. It would be interesting to understand how performing an active response task over the same time period affects image quality, pupil size and defocus in comparison to current results, particularly when the time point of stimulus onset is not known to the participant. Fourth, it would be useful to record aberration measurements from the SHWS in future studies to analyse the effect of residual aberrations (defocus and higher-order aberrations) and how they are affected by both the size and location of the pupil over an extended period. Last but not least, it would have been ideal to run the same study with pupil dilation and compare between results. We are keen to make this comparison once we receive the necessary approval.

## 5. Conclusion

In summary, through statistical analysis we have demonstrated the feasibility of performing stable and good quality AOSLO imaging at $4^{\circ }$ eccentricity over 1.5 hours without pharmacological pupil dilation. Experimental results showed that image quality strongly correlates with pupil size. MTF modelling further suggested that pupil size is not the only factor affecting image quality for an undilated pupil. A dim and steady background is recommended for imaging with a natural pupil, while reliable imaging closer to the fovea would benefit from pupil dilation. These results are particularly important when dilating drops are contraindicated for medical or research reasons.

## Data Availability

Data underlying the results presented in this paper are not publicly available at this time but may be obtained from the authors upon reasonable request.
